# Clinical Outcomes Following Posterior Malleolus Fixation in Trimalleolar Ankle Fractures: A Prospective Study

**DOI:** 10.5704/MOJ.2511.012

**Published:** 2025-11

**Authors:** SD Chaudhary, KR Lakhani, S Andhale, A Sonkusale

**Affiliations:** 1 Department of Orthopaedics, Government Medical College, Nagpur, India; 2 Department of Orthopaedics, Indira Gandhi Government Medical College and Hospital, Nagpur, India; 3 Department of Orthopaedics, Apollo Hospitals, Chennai, India

**Keywords:** posterior malleolus, trimalleolar fractures, posterolateral, posterior malleolar fixation

## Abstract

**Introduction:**

Posterior malleolus (PM) fractures are frequently caused by pronation or supination injuries with an external rotation component to the ankle. Historically, fixation of the PM was not considered essential if it involved less than 25% of the tibial articular surface. However, studies have now shown that trimalleolar fractures fare worse than bimalleolar fractures. This study primarily aims to evaluate the clinical outcomes of trimalleolar fractures, focusing on posterior malleolus fixation regardless of the fragment size.

**Materials and methods:**

A prospective observational study was undertaken to investigate the efficacy of PM fixation at a tertiary care centre from October 2020 to December 2022. All participants underwent pre-operative radiographs and were classified according to the Lauge-Hansen system. Sixteen consecutive patients underwent the posterolateral approach to reduce and stabilise the PM, utilising either a buttress/antiglide plate or a posterior-to-anterior (PA) screw, in conjunction with fixation of the medial and lateral malleoli. Patient characteristics, injury specifics, surgical details, and complications were documented. Clinical outcomes were assessed using the American Orthopaedic Foot and Ankle Society (AOFAS) scoring system.

**Results:**

The study cohort included 16 patients with an average follow-up of 18 months. The AOFAS scores indicated excellent outcomes in six cases, good outcomes in eight cases, and a fair outcome in two cases. PA lag screw fixation was used in seven patients when the fracture fragment was large enough with three excellent, three good and one fair outcome. While buttress/antiglide plate fixation was employed in nine patients when the fracture fragment was small or comminuted with three excellent, five good and one fair outcome. One patient developed a superficial infection, which was managed with debridement, and another patient experienced malunion. Both of these patients had a fair outcome.

**Conclusion:**

A posterolateral approach allows fixation of the posterior malleolus and fibula through a single incision, ensuring anatomical reduction and stable fixation. This method yields excellent outcomes with minimal complications, though further research with larger studies is needed.

## Introduction

Ankle fractures constitute about 10% of all fractures, making them the second most common fracture of the lower limb after hip fractures^1,2^. Of these fractures, posterior malleolus (PM) fractures involve 14 – 40% of ankle fractures^3^. While often overlooked, the posterior malleolar fracture fragment (PMFF) is an important contributor to stability in trimalleolar fractures. Ankle fractures that involve the PM tend to have significantly poorer clinical outcomes compared to those that do not affect this area^4,5^.

Management of PM fractures has always been a topic of debate with no clear consensus on the necessity or method of fixation. Nelson and Jensen introduced the ‘one-third rule’ which considered PM fractures significant only if they involved more than 1/3rd of the tibial plafond^6^. Subsequently, in recent times contribution of the PM to articular congruence along with posterior syndesmotic stability has gained significance over the size of the PMFF, this necessitates a CT scan for accurate evaluation of the fracture pattern^7^. Preserving an intact PM with the attached posterior inferior tibiofibular ligament (PITFL) is essential to prevent secondary displacement and shortening of the fibula^8^.

Over the years, numerous studies have consistently demonstrated that operative fixation of the posterior malleolus in trimalleolar fractures results in superior postoperative functional outcomes^9-11^. Despite this, many orthopaedic surgeons continue to rely on the size of the posterior malleolar fragment as a key criterion for deciding whether to proceed with surgical intervention^12^. The aim of this study is to show that fixation of the posterior malleolus in trimalleolar fractures can lead to excellent functional outcomes, regardless of the size of the fracture fragment.

## Materials and Methods

We conducted a prospective study after receiving clearance and approval from the institutional ethics committee and obtaining written informed consent from patients who agreed to undergo the surgical procedure. The study included skeletally mature patients with closed trimalleolar ankle fractures who presented within 14 days of injury. Patients with previous malunion or non-union at the ankle, neurovascular injury, pathologic fracture, open injuries, and non-ambulatory patients with neuromuscular disorders were excluded from the study. All patients were evaluated with pre-operative radiographs and computed tomography scans.

**Fig. 1:**
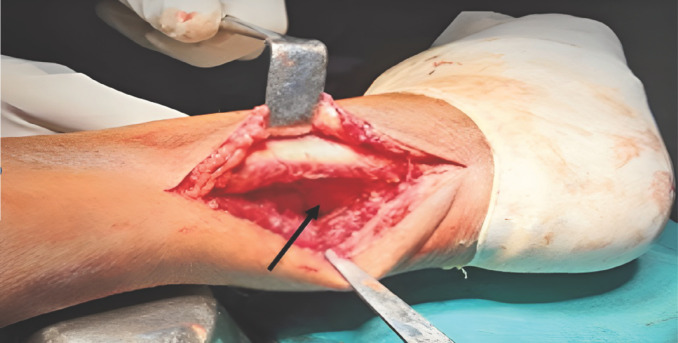
Posterolateral approach with direct visualisation of the PM fragment (black arrow).

**Fig. 2:**
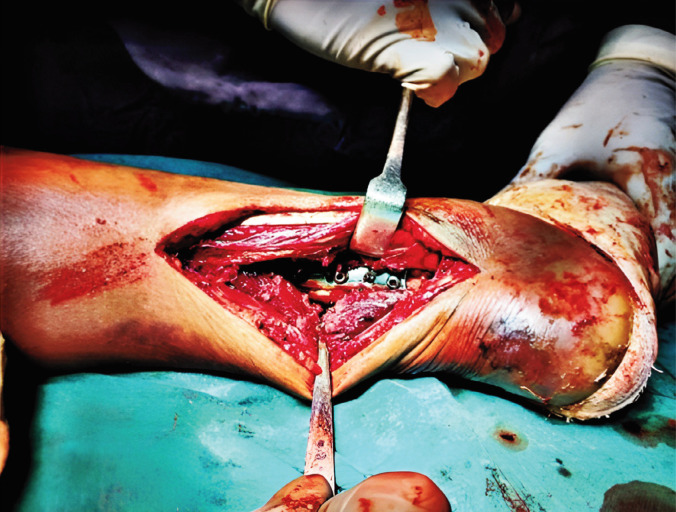
Buttress plate applied over the posterior malleolus.

**Fig. 3:**
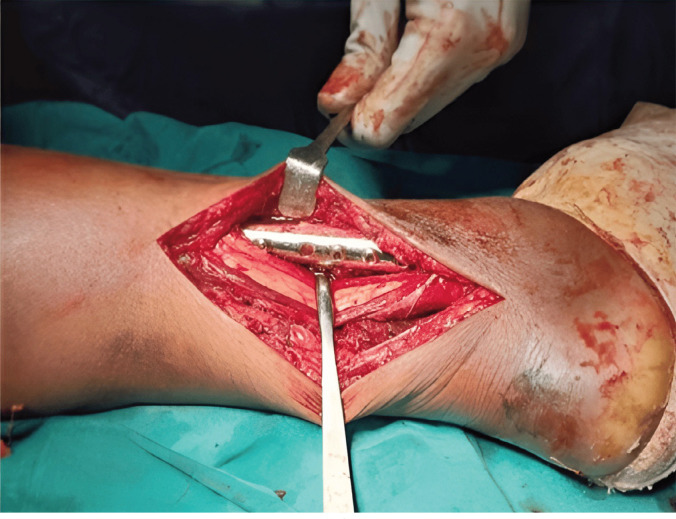
Using the same approach the lateral malleolus is fixed with a 1/3rd tubular plate.

All procedures were performed under spinal or general anaesthesia. With the patient in a lateral decubitus position, we used the posterolateral approach (Fig. 1) to expose both the PMFF and the fibular fracture. The PMFF was addressed first, it was reduced under direct vision and fixed with a buttress/antiglide plate in cases where the fragment was small or comminuted (Fig. 2). In large fragments, we used at least two cannulated cancellous screws to fix the fracture in a posteroanterior direction. Using the same incision the fibular fracture was exposed and fixed with a plate positioned on the posterior aspect of the fibula (Fig. 3). Posterior plating was utilised as it allows the plate to be used as an antiglide plate and prevents soft tissue complications commonly associated with lateral plating of the fibula^13^. The patient was then repositioned to a supine position, and a medial incision was utilised to address the medial malleolus. Post-operatively, all patients were kept non-weight bearing for six weeks and the ankle was protected in a below-knee backslab for two weeks. At two weeks ankle movements were permitted in a graded manner under supervision following suture removal.

All patients were kept non-weight bearing on the operated limb and mobilised with a walker for six weeks. Toe touch weight bearing was allowed at six weeks. Full weight bearing was allowed after radiological and clinical signs of union. All patients were evaluated radiologically by serial radiographs taken on post-operative day 0, 6 weeks, 12 weeks and then at 3 monthly intervals henceforward. Functional evaluation was done using the American Orthopaedic Foot and Ankle (AOFAS) score at every follow-up.

## Results

During the study period, a total of 47 ankle fractures were admitted and treated at our institute. Of these, 16 cases met the inclusion criteria and were incorporated into the study, with a mean follow-up duration of 18 months (range: 15 to 20 months). The cohort consisted of 12 male patients and 4 female patients, with a mean age of 41 years (ranging from 22 to 65 years). Of the 16 patients included in our study the mode of injury in 50% (8/16) patients was road traffic accidents where as in 18.75% (3/16) patients it was a fall from height and 31.25% (5/16) patients had a twisting injury. There was significant soft tissue swelling in 81.25% (13/16) of these patients, and in all of these patients the operative intervention was delayed by 7-10 days to allow time for the soft tissue condition to improve. The majority of patients, 62.5% (10/16), were between the ages of 18 and 40 years (Table I). In 18.75% (3/16) of patients the time to surgery was withing 7 days from injury, in 75% (12/16) of patients the time to surgery was between 8 to 14 days from injury and in 6.25% (1/16) of patients the time to surgery was between 15-21 days from surgery (Table II).

**Table I:** Demographic characteristics.

Variable	Number
Sample Size	16
Mean age (in years)	41
Sex (male : female)	12 : 4
Side (right / left)	9 / 7
Mechanism of injury	
Road traffic accidents	8
Twisting injuries	5
Fall from height	3

**Table II:** Time to surgery.

Days from injury	Number of patients
0 – 7	3
8 – 14	12
15 – 21	1

**Fig. 4:**
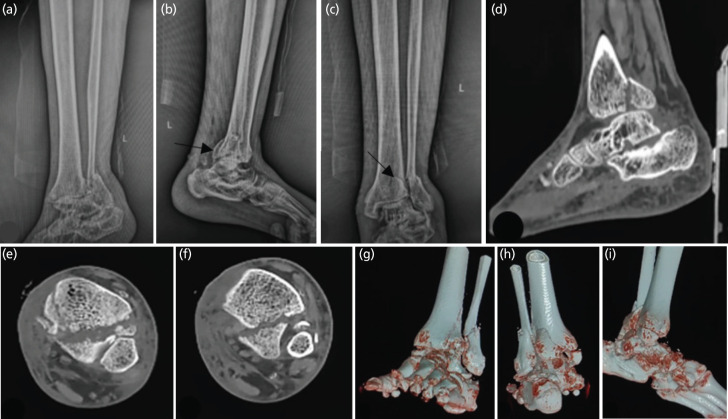
Pre-operative radiographs and CT of a 56-year-old male patient; (a) shows anteroposterior view radiograph of the ankle, we can identify the fractured lateral and medial malleolus; (b) shows lateral view radiograph, here we can identify the posterior malleolar fracture fragment (arrow); (c) shows a mortise view radiograph of the ankle in which the fracture pattern is better appreciated and the posterior malleolar fracture fragment appears to be posterolaterally located (arrow); (d) shows the sagittal cut CT scan 2D image of the ankle; (e) shows the axial cut CT scan 2D image of the ankle here we can identify that the posterior malleolar fracture fragment involves the whole of the posterior malleolus distally; (f) shows another axial cut CT scan 2D image of the ankle which is a few cms proximal to the previous one; (g, h, i) show 3D reconstructed images of the ankle showing a posterolateral posterior malleolar fracture fragment along with a transverse medial malleolar fracture and an oblique lateral malleolar fracture.

**Fig. 5:**
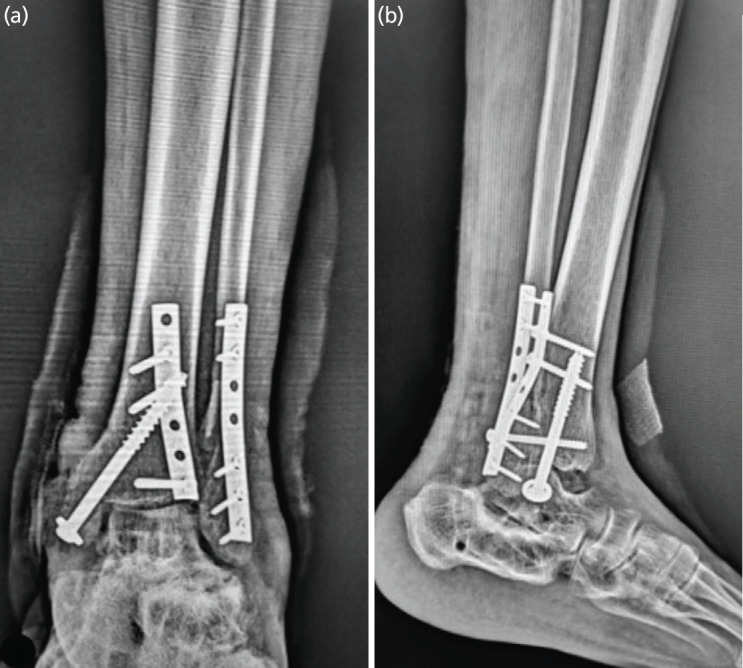
Post-operative radiographs of the same patient; (a) shows the anteroposterior view showing the posterior malleolus fracture fragment fixed with a semitubular plate in buttress mode; (b) shows the lateral view of the same.

Regarding the injury types, 62% (10/16) had Supination-External Rotation (SER) injuries, 19% (3/16) had Pronation-External Rotation (PER) injuries, 13% (2/16) had Supination-Adduction (SAD) injuries, and 6% (1/16) had Pronation-Abduction (PAB) injuries. Treatment varied, with 56.25% (9/16) of patients managed with buttress plate fixation and 43.75% (7/16) treated with posterior-anterior (PA) screw fixation. Outcomes were largely favourable, with 87% (14/16) of patients achieving excellent to good results, while 13% (2/16) had fair outcomes (Table III). In terms of sagittal motion (flexion and extension), 75% (12/16) of patients demonstrated either normal or mild restriction according to the AOFAS score, 18.75% (3/16) experienced moderate restriction, and 6% (1/16) had marked restriction. Fig. 4 shows the radiographic images while Fig. 5 shows the post-operative radiographs of a representative case in which the posterior malleolus was fixed using a buttress plate.

**Table III:** AOFAS and distribution according to mode of fixation.

	AOFAS				Total
	Excellent	Good	Fair	Poor	
**Mode of Fixation**						
Buttress plating	3	5	1	0	9 (56.25%)
Postero-anterior screw	3	3	1	0	7 (43.75%)
Total	6 (37%)	8 (50%)	2(13%)	0	

One patient in the buttress plating group experienced a superficial surgical site infection which was managed with intravenous antibiotics and debridement with secondary suturing following which the wound went on to heal well and one patient in the PA screw fixation group had malunion due to loss of reduction on follow-up radiographs. Both of these patients had a fair outcome.

## Discussion

Over the past few years, posterior malleolus fractures have gained importance with regard to post-operative ankle stability and joint congruity in an attempt to improve the functional outcome of trimalleolar fractures and prevent post-operative osteoarthritis of the ankle. Although the primary restraint to posterior forces is the Anterior inferior tibio-fibular ligament (AITFL), posterior inferior tibio-fibular ligament (PITFL) and the fibula, the PM is an important stabiliser to control the instability of the ankle^14.^ Most PMFFs tend to be small, laterally based fragments, still attached to the PITFL^15^.

Whether or not to fix the PMFF in a trimalleolar fracture and the method of fixation has been a topic of debate in the literature^4,15-17^. While some surgeons still rely on fragment size as one of the main indications for fixation with thresholds for surgery ranging from one-fourth to one-third of the anteroposterior dimension of the articular surface^6^. Others believe that in complex ankle or tibial plafond injuries associated with displaced fractures of the PM, reduction and fixation of the fragment is essential for the restoration of joint mechanics^18-22^. Most current studies suggest that the PM is a direct extension of the PITFL, and the size of the fragment should not be the sole criteria for fixation. Other factors like articular step-off, plafond impaction and intercalary fragments should dictate the plan of management in these fractures^8,17,20^.

Although most of the PMFFs encountered by orthopaedic surgeons are large with a single triangular posterolateral fragment, Haraguchi *et al* in 2006 described three different types of PMFFs, posterolateral-oblique (type-I)- a wedge-shaped fragment involving the posterolateral corner of the tibial plafond, transverse medial-extension (type-II) fractures- fracture line extending from the fibular notch of the tibia to the medial malleolus, and small-shell (type-III) fractures - having one or more small shell-shaped fragments at the posterior lip of the tibial plafond. Their study advised the use of computed tomography scans to analyse the PMFFs and plan the surgical approach accordingly^7^. Fixation of the PMFFs potentially reduces the need for direct syndesmosis stabilisation^3,19^.

There have been various studies on the method of fixation to be used for fixing the posterior malleolus in trimalleolar fractures. Historically a minimally invasive approach involving anteroposterior screw fixation of the posterior malleolus without formal open reduction was popular to prevent soft tissue complications, reduce operative time and preserve the biological soft tissue cover over the ankle. However, since the reduction and screw trajectory is judged on the basis of fluoroscopic images, the reduction may not be optimal and the screws may not be engaging the fragment well. Over the years the majority of surgeons have moved from the former method to a more direct approach with either posterolateral or posteromedial extensile exposures of the posterior malleolus and open reduction along with either Antiglide plate fixation or posteroanterior screw fixation^23-26^. This method enables the surgeon to achieve anatomical restoration of the distal tibiotalar joint while also providing a more stable construct. We found in our study that approaching the posterior malleolus fracture fragment via the posterolateral approach not only provides ease of reduction but also allows for the fibula to be stabilised through the same incision^27^.

Bennett *et al* in their 2016 study concluded that buttress/antiglide plating in PM fractures provided a significantly better construct with minimal displacement on cyclical loading^23^. While Zhang *et al* in their comparative study concluded that PA lag screws or a posterior buttress plate through the posterolateral approach both showed good and equivalent clinical and radiological outcomes with minimal complications^28^. However, we based the implant choice based on fracture type and the amount of comminution, for fractures with large fragments and minimal comminution, we opted for direct under-vision reduction followed by posteroanterior cannulated cancellous screw fixation. In contrast, for small fragments and those with comminution, we preferred buttress/antiglide plating with a small fragment plate.

The clinical outcomes were calculated using the AOFAS questionnaire which consists of a questionnaire examining pain (40 points), function in daily living (28 points), range of motion (22 points), and ankle alignment (10 points). At the end of the 6-month follow-up, the mean AOFAS score was 90.43 indicating an excellent outcome which is quite similar to other studies (Table IV)^25,29-32^.

**Table IV:** Outcome comparison with other studies in literature.

Studies	Year	Country	Mean AOFAS
Zhong S *et al*^29^	2017	China	89.9
Fidan F *et al*^30^	2021	Turkey	91.6
Taki M *et al*^25^	2021	Japan	93
Neumann AP *et al*^31^	2022	Germany	87.5
Sun C *et al*^32^	2022	China	88.6
Our study	2025	India	90.43

This study aims to demonstrate that fixation of the posterior malleolus using the posterolateral approach in all cases of trimalleolar fractures can result in excellent functional outcomes with minimal complications. Additionally, this technique allows for simultaneous fixation of the lateral malleolus through the same incision.

The limitations of our study are the absence of a control group with anteroposterior screw fixation for comparison, a non-operative control group and a relatively small cohort.

## Conclusion

In summary, Excellent outcomes can be achieved with the posterolateral approach for posterior malleolar fixation with minimal complications. However, more research is needed on this and comparative studies with a larger sample size would provide further clarity on the management of posterior malleolar fractures.
